# Niacin alters ruminal microbial composition and metabolites in sheep fed a high-concentrate diet

**DOI:** 10.3389/fvets.2025.1510617

**Published:** 2025-01-28

**Authors:** Zhiqiang Cheng, Jiancheng Liu, Yingying Yu, Wentao Liu, Xiaobin Li, Fengming Li, Changjiang Zang, Kailun Yang

**Affiliations:** ^1^College of Animal Science, Xinjiang Agricultural University, Ürümqi, China; ^2^Xinjiang Ürümqi Rural Revitalization Guidance Service Center, Ürümqi, China

**Keywords:** growth performance, rumen fermentation, rumen microbial community, metabolism, niacin, sheep

## Abstract

This study aimed to investigate the effects of niacin supplementation to a high-concentrate diet (ratio of concentrate supplement to forage = 70:30) on the growth performance, rumen fermentation, rumen microbiota, and metabolomics of sheep. Twelve sheep were randomly divided into two groups: (1) a control group (CON, *n* = 6) fed a basal diet and (2) a niacin group (NA, *n* = 6) fed a basal diet supplemented with 130 mg/day niacin for 35 days: days 1–14 were the adaptation period, days 15–35 were the experiment period. On days 15 and 35 of the experiment period, all trial sheep were weighed before the morning feed (07:30 am). Ruminal fluid samples were collected from all trial sheep on days 34 and 35. The results showed that (1) the dry matter feed intake of the NA group was higher than that of the CON group (*p* < 0.05). (2) The ruminal pH of the NA was significantly higher than that of the CON group at 3, 5, and 7 h after feeding (*p* < 0.01). The concentrations of NH_3_-N (*p* < 0.01), propionate (*p* < 0.01), and butyrate (*p* < 0.05) in the NA group were significantly higher than those in the CON group. (3) Compared to the CON group, the ruminal pyruvate content in the NA group was significantly increased at 0 h before feeding (*p* < 0.05), and lactic acid (*p* < 0.05) was significantly decreased at 1 and 3 h after feeding, lactate dehydrogenase activities was significantly decreased (*p* < 0.01) at 3 and 5 h after feeding. (4) The number of specific operational taxonomic units (OTUs) in the CON and NA groups were 26 and 37, respectively, for a total of 1,178 OTUs; principal coordinate analysis (*R*^2^ = 0.172, *p*-value = 0.007) and non-metric multidimensional scaling (stress = 0.1646) results showed that the two groups of samples were significantly separated. (5) The species distribution bar graph shows that at the phylum level, the relative abundances of Bacteroidetes, Firmicutes, and Proteobacteria were 43.70, 36.25, and 12.77%, respectively. (6) Orthogonal projection to latent structure-discriminant analysis results showed that the two groups of samples were clearly separated in the positive and negative ionization modes, with *R*^2^*Y* and *Q*^2^*Y* values of 0.705, 0.857, 0.695, and 0.28, respectively. There were 72 metabolic pathways, mainly citric acid cycle, pyruvate metabolism, and cysteine and methionine metabolism. (7) Correlation analysis showed that a number of microorganisms (such as *Succinivibrio* and *Prevotella*) and differential metabolites (such as L-malic acid, propionic acid, succinic acid, and pyruvic acid) participated in tricarboxylic acid cycle metabolism. In summary, supplementing niacin to high-concentrate diets can significantly improve the growth performance of sheep, improve rumen fermentation and the rumen microbial community structure, and affect rumen metabolites, thus alleviating the symptoms of rumen acidosis.

## 1 Introduction

In recent years, high-yield dairy cows and finishing sheep have been fed high-grain diets to improve their growth performance. Yet the excessive intake of starch-enriched high-grain diets causes volatile fatty acid (VFA) accumulation and lactate production in the rumen (1). VFA accumulation in the rumen along with microbial fermentation can increase lactic acid production and rapidly decrease pH (2–4), leading to rumen acidosis. High concentrations of VFA can also disrupt the rumen microbial community, changing its diversity and balance and severely affecting the proliferation and development of bacteria. This results in a decrease in the number and abundance of bacteria (5), especially in regards to the phylum Bacteroidetes (6). Furthermore, over consumption of a high-grain diet can increase the number of starch-degrading bacteria, such as *Lactobacilli* and *Selenomonas ruminantium*, in the rumen; the number of *Lactobacillus* spp., in particular, has been observed to significantly increase in this situation (7). Such changes in the rumen microbiome, particularly in the bacterial flora, alter ruminal metabolic pathways and patterns, ultimately affecting the health status of the animal.

Niacin is the precursor of the coenzymes NAD^+^ and NADH and plays a crucial role in the metabolism of carbohydrates, proteins, and lipids. Dietary supplementation with niacin improves growth performance (8) as well as the rumen environment by promoting the growth of microorganisms while inhibiting the proliferation of lactate-producing bacteria (*Streptococcus bovis*), which reduces the accumulation of lactate (9). Supplementation with niacin also improves fermentation in the rumen, which can affect the diversity and relative abundance of rumen flora and regulate the dynamic balance between lactic acid and lactic-utilizing bacteria, thereby regulating lactate production (10). Our laboratory previously conducted research on the effects of niacin supplementation (100, 130, and 160 mg/day) on growth performance, digestive metabolism, and rumen fermentation. The results showed that 130 mg/day of niacin had the greatest effect (11, 12). Therefore, in this study, the high-concentrate diets of sheep were supplemented with 130 mg/day of niacin and then 16S rRNA sequencing combined with metabolomics was used to explore the effects of this supplementation on specific differential microbial metabolites and changes in rumen microorganisms. The results of this study provide new insights into the healthy breeding of ruminants fed high-concentrate diets.

## 2 Materials and methods

### 2.1 Ethics statement

All experimental protocols were approved by the Animal Care Committee of Xinjiang Agricultural University (No. 2020024).

### 2.2 Experimental design and diets

Twelve Kazakh rams with rumen fistula [location: Changji, China; age: 8 months old; average initial body weight (BW): 36.09 ± 1.81 kg] were selected for this study. The sheep were randomly allocated to either the CON group (*n* = 6) or NA group (*n* = 6). Sheep in the CON group were fed a basal diet (concentrate supplement to forage ratio 70:30), and sheep in the NA group were fed a basal diet that was supplemented with 130 mg/day niacin (Shanghai Yuanye Biotechnology Co., Ltd., Shanghai, China). The experiment lasted for 35 days: days 1–14 were the adaptation period, days 15–35 were the test period. Basal diets were formulated according to the Meat Sheep Feeding Standards (NY/T816-2021) (Table 1).

**Table 1:** Composition and nutrient levels of the basal diet (dry matter-based).

Item	Content
Ingredients, %	
Corn stalk	21.00
Alfalfa	9.00
Corn	35.00
Wheat bran	8.40
Soybean meal	14.00
Cottonseed meal	9.10
Premix^a^	3.50
Total	100.00
Chemical composition, %	
Dry matter (DM)	91.33
Crude protein (CP)	17.13
Ether extract (EE)	1.91
Cellulose (CEL)	13.59
Hemicellulose (HC)	9.71
Lignin	3.31
Ca	1.02
P	0.55
ME (MJ/kg)^b^	14.12

DM, dry matter; CP, crude protein; EE, ether extract; CEL, cellulose; HC, hemicellulose; ME, metabolic energy.

a The premix provided the following per kg of diets: VA 7,000 IU, VD3 1,785 IU, VE 14 IU, biotin 0.04 mg, Cu (as copper sulfate) 8.8 mg, Fe (as ferrous sulfate) 26.32 mg, Mn (as manganese sulfate) 29.14 mg, Zn (as zinc sulfate) 35.53 mg, I (as potassium iodide) 0.57 mg, and Se (as sodium selenite) 0.23 mg.

b ME was a calculated value, while the others were measured values.

### 2.3 Sample collection

During the experiment, dry matter intake (DMI) and BW (determined at 07:30 am, before the morning feeding) were recorded. All samples were oven dried at 65°C for 48 h to a constant weight. The dried samples were ground and passed through a 1-mm sieve for subsequent analysis.

On days 15 and 35 of the experiment period, all trial sheep were weighed before the morning feed (07:30 am). Ruminal fluid samples were collected from all trial sheep on days 34 to 35 of the experiment. All ruminal fluid samples were collected 0 h before morning feeding and 1, 3, 5, and 7 h after feeding from six sheep (three sheep in the CON group and three sheep in the NA group) every day.

To avoid salivary contamination, 80 mL of ruminal fluid was collected through the ruminal fistula and filtered through four layers of gauze. The first 30 mL of the ruminal fluid was discarded, leaving 50 mL. Ruminal pH was immediately measured using a portable pH meter (Anscitech Co., Ltd., Wuhan, Hubei, China), and ruminal fluid pH was measured 0 h before morning feeding and 1, 3, 5, and 7 h after feeding. Fifteen milliliters of ruminal fluid were squeezed through four layers of cheesecloth, transferred into sterile tubes, and stored at −20°C for subsequent analysis. A total of 12 sheep ruminal fluid samples were collected in this study.

### 2.4 Sample analysis and calculation

Each sample was analyzed for dry matter (DM; method No. 930.15) by drying at 105°C to a constant weight, ether extract (EE; method No. 989.05) using Soxhlet extraction with diethyl ether and crude protein (CP; method No. 976.05) using the Kjeldahl method with a Buchi Distillation Unit B-324 (Büchi Labortechnik AG, Flawil, Switzerland), as described by the Association of Official Analytical Chemists (13). Cellulose, hemicellulose, and lignin contents were determined according to the Van Soest method (14) using fiber bags and an ANKOM 220 Fiber Analyzer (ANKOM Technology Company, Macedonia, NY, United States).

Frozen ruminal fluid samples were thawed and centrifuged at 12,000 rpm for 10 min at 4°C to obtain a clear supernatant. Then, 1 mL of the supernatant was mixed with 0.25 mL of a metaphosphoric acid standard solution (25 g/100 mL) for subsequent analysis. The concentration of ammonia-N (NH_3_-N) was determined using the phenol-sodium hypochlorite colorimetric method described by Broderick and Kang using a UV spectrophotometer (UV-1801, Beijing Beifen Ruili Analytical Instrument Co., Ltd., Beijing, China) (15). The VFA concentrations were analyzed using gas chromatography according to the method described by Mirzaei-Alamouti et al. (16). The VFA was quantified using a high-performance gas chromatograph (GC-2014; Shimadzu Corporation, Kyoto, Japan) that was equipped with a hydrogen flame detector and capillary column (Agilent Technologies, Santa Clara, CA, United States; 30 m long, 0.32-mm diameter, 0.50-μm film thickness). The remaining liquid samples were then immediately frozen in liquid nitrogen, transported to the laboratory, and stored at −80°C for further analysis of microbiota and metabolites. The intermediate products of lactic acid metabolism and lactate metabolic enzyme activity in the rumen fluid were determined based on samples taken at 0 h before feeding and at 1, 3, and 5 h after feeding. Ruminal lactate metabolism intermediates were determined using liquid chromatography (LC-20ADXR; Shimadzu Corporation, Kyoto, Japan), and related enzymes were determined using kits (Nanjing Jiancheng Bioengineering Institute, Nanjing, China).

### 2.5 Extraction and sequencing of DNA

DNA was extracted from ruminal fluid samples using a DNA extraction kit, and the V3–V4 region of the 16S rRNA gene in the conserved region was amplified. Samples with a bright main strip were chosen. The amplified length was approximately 419 bp (Biomarker Technologies, Rohnert Park, CA, United States). Samples were then sequenced on an Illumina NovaSeq 6000 (Illumina, San Diego, CA, United States) and subjected to quality inspection; high-quality raw data were trimmed. The decontamination-optimization process yielded effective data. The 97% similarity level was clustered using USEARCH software to obtain operational taxonomic units (OTUs) (17). Sequences were diluted 50,000-fold and normalized. Alpha diversity analysis was performed using the Mothur software^1^ to calculate species richness and diversity (18) using the Chao1, Ace, Shannon, and Simpson indices and distance matrices of samples obtained using weighted and unweighted algorithms. Principal component analysis (PCA), principal coordinate analysis, and significant species difference analysis were performed using QIIME software with an unweighted UniFrac distance matrix for the principal coordinate analysis.

### 2.6 Metabolomics data analysis

The ruminal fluid samples were thawed, further ground, and extracted, and metabolites were extracted into the extraction solution (mobile phase methanol-acetonitrile, volume ratio of 1:1), sonicated for 10 min, and then kept at −20°C under static conditions. After 1 h, the samples were centrifuged at 12,000 rpm for 15 min at 4°C. Subsequently, 500 μL of the samples was placed into an Eppendorf tube and dried in a vacuum concentrator for 10 min, and 160 μL of extraction solution (acetonitrile-water volume ratio 1:1) was added to the dried metabolite to reconstitute it. It was vortexed for 30 s, placed in an ice-water bath and sonicated for 10 min, and then centrifuged at 4°C at 12,000 rpm for 15 min. Next, 120 μL of supernatant was carefully removed and placed into a 2-mL injection vial, and 10 μL of each sample was mixed into quality control samples for onboard detection (19). The liquid chromatography/mass spectrometry (LC-MS) system for metabolomic analysis consisted of a Waters Acquity-Class Plus ultra-high-performance liquid chromatograph (Waters, Milford, MA, United States) in series with a Waters Xevo G2-XS QTOF high-resolution mass spectrometer (Waters) and Waters Acquity-UPLC HSS T3 column (1.8 μm 2.1*100 mm) (20). The positive ion mode comprises a mobile phase A containing 0.1% formic acid aqueous solution and a mobile phase B containing 0.1% formic acid acetonitrile. The negative ion mode comprises a mobile phase A containing 0.1% formic acid aqueous solution and a mobile phase B containing 0.1% formic acid acetonitrile. The injection volume was 1 μL.

The Waters Xevo G2-XS QOF high-resolution mass spectrometer (Waters) can perform primary and secondary mass spectrometry data acquisition in the MSe mode under the control of an acquisition software (Mass Lynx V4.2; Waters). Dual-channel data acquisition for low collision energies can be performed simultaneously in each data acquisition cycle. The low collision energy was 2 V, the high collision energy range was 10–40 V, and the scanning frequency was 0.2 s for a mass spectrum. The ESI ion parameters were as follows (21): capillary voltage, 2,500 V (positive ion mode) or −2,000 V (negative ion mode); cone voltage, 30; Vion source temperature, 100°C; desolvation gas temperature, 500°C; flow rate of backflushing gas, 50 L/h; flow rate of desolventizing gas, 800 L/h; and collection range of mass-to-nucleus ratio, 50–120.

### 2.7 Statistical analysis

Analysis of variance was performed using SPSS software (version 27.0; SPSS Institute Inc., Cary, NC, United States). For initial body weight, final body weight, average daily gain, DMI, pH, NH_3_-N, and VFA, *t*-tests were used to analyze the differences between the microbiome groups, and Spearman’s correlation was performed for the top 20 flora. The correlation between rumen microorganisms and rumen metabolites [variable importance in projection (VIP) >1, fold change (FC) >1 or (FC) <1, and *p* < 0.05] was evaluated; the data shown represent the mean and standard error of the mean. Statistical significance was set at *p* < 0.05 or *p* < 0.01. The R programming language was used to draw principal coordinate analysis (PCoA), Venn, non-metric multidimensional scaling (NMDS), and correlation analysis diagrams. The α-diversity index was plotted using GraphPad Prism 10.0.

## 3 Results

### 3.1 Growth performance

As shown in Table 2, there were no significant differences in the initial BWs, final BW, and average daily gain between the CON and NA groups (*p* > 0.05), whereas the DMI (*p* < 0.05) in the NA group increased significantly when compared to that of CON group.

**Table 2:** Effects of niacin on growth performance and rumen fermentation parameters.

Items	CON	NA	SEM	*p*-value
Initial BW (kg)	36.07	36.10	1.08	0.866
Final BW (kg)	41.23	43.52	0.88	0.200
DMI (g/day)	1484.67	1615.00	43.05	0.044
ADG (g/day)	214.83	274.17	8.05	0.218

DMI, dry matter intake; ADG, average daily gain; CON, control group (basal diet); NA, niacin group (basal diet + 130 mg/day niacin); SEM, standard error of the mean. *p* < 0.05 indicates a significant difference.

### 3.2 Rumen fermentation parameters

**Figure 1:**
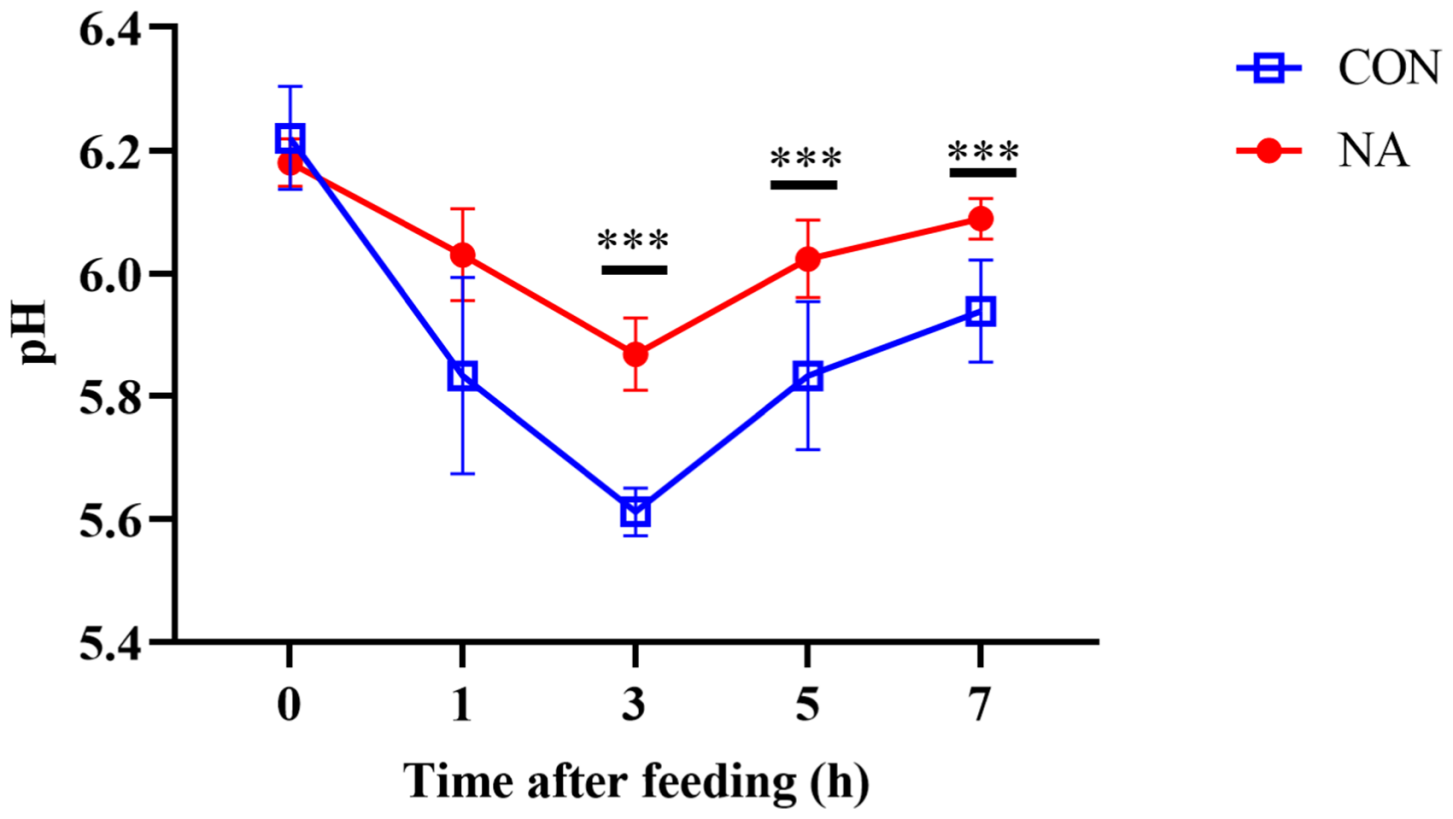
Effects of niacin on ruminal pH. The measured ruminal fluid pH of the CON group and the NA group of sheep collected at 0 h before feeding and 1, 3, 5, and 7 h after feeding on the 34th and 35th days of the experiment. A single asterisk (*) indicates a significant difference (*p* < 0.05), a double asterisk (**) or triple asterisk (***) indicate highly significant differences (*p* < 0.01).

**Figure 2:**
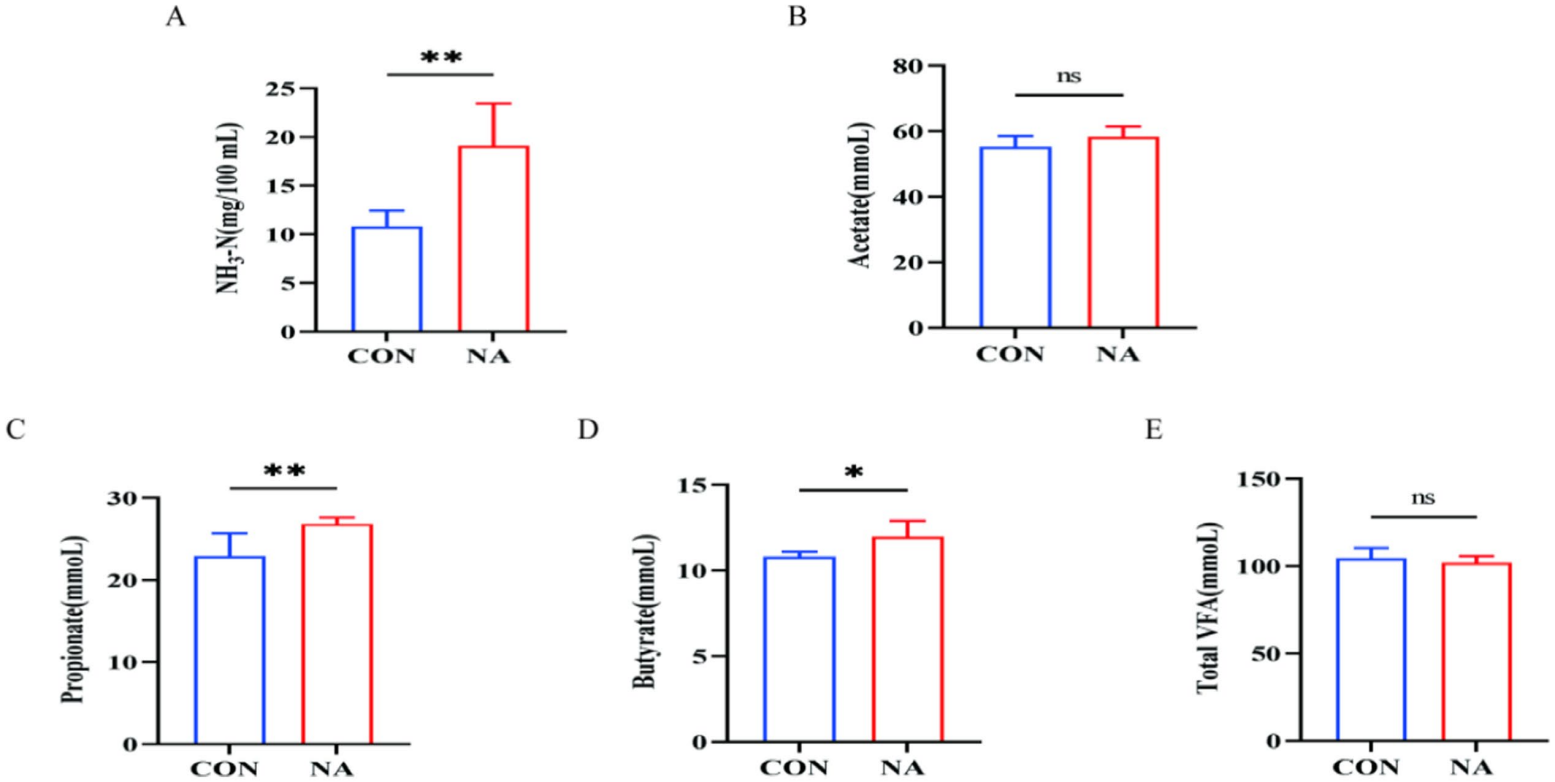
Effects of niacin on rumen fermentation parameters. NH_3_-N, ammonia-N; total VFA, total volatile fatty acid; CON, control group (basal diet); NA, niacin group (basal diet + 130 mg/day niacin). **(A)** Ammonia nitrogen. **(B)** Acetate. **(C)** Propionate. **(D)** Butyrate. **(E)** Total volatile fatty acids. The results are presented as the mean and standard error. A single asterisk (*) indicates a significant difference (*p* < 0.05), while a double asterisk (**) indicates a highly significant difference (*p* < 0.01), *t*-test, *n* = 6.

Compared to the CON group, ruminal fluid pH in the NA group increased significantly at 3, 5, and 7 h after feeding (*p* < 0.01) (Figure 1). As shown in Figure 2, the NH_3_-N (*p* < 0.01), propionate (*p* < 0.01), and butyrate (*p* < 0.05) concentrations were significantly higher in the NA than those in the CON group. No significant differences were observed in acetate and total VFA concentrations in the rumen between the two groups (*p* > 0.05).

### 3.3 Metabolic enzymes and products

**Figure 3:**
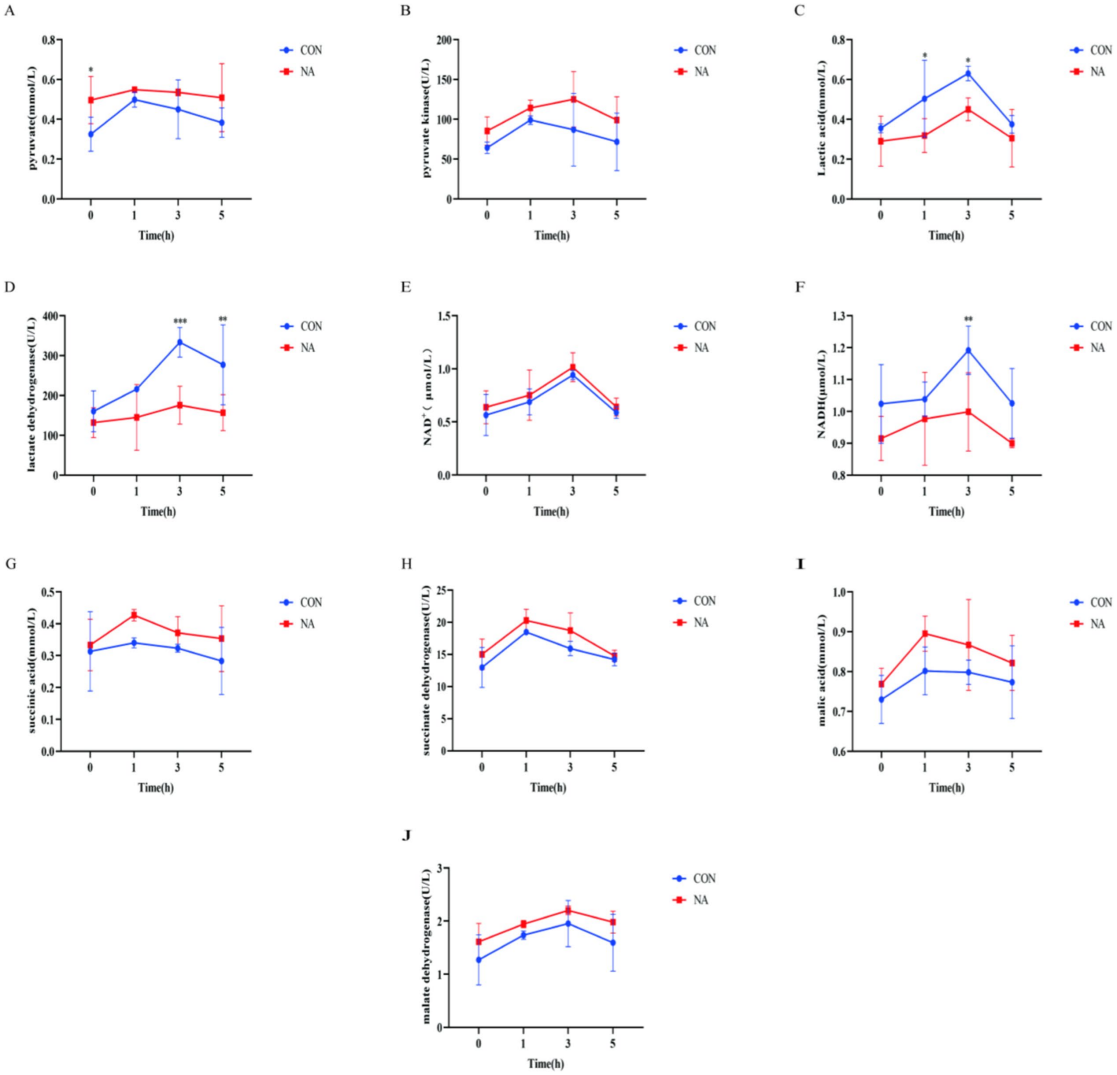
Ruminal metabolites and related enzymes. CON, control group (basal diet); NA, niacin group (basal diet + 130 mg/day niacin). **(A)** Pyruvate. **(B)** Pyruvate kinase activity. **(C)** Lactic acid. **(D)** Lactate dehydrogenase activity. **(E)** Oxidized nicotinamide adenine dinucleotide. **(F)** Reduced nicotinamide adenine dinucleotide. **(G)** Succinic acid. **(H)** Succinate dehydrogenase. **(I)** Malic acid. **(J)** Malate dehydrogenase activity. The results are presented as the mean and standard error. A single asterisk (*) indicates a significant difference (*p* < 0.05), a double asterisk (**) or triple asterisk (***) indicate highly significant differences (*p* < 0.01), *t*-test, *n* = 6.

As shown in Figure 3, all indicators such as pyruvate content (Figure 3A), pyruvate kinase activity (Figure 3B), and lactate content (Figure 3C) in the CON group and the NA group showed a trend of first increasing and then decreasing as the time after feeding prolonged. Compared with the CON group, the pyruvate content (Figure 3A) of the NA group was significantly increased 0 h before feeding (*p* < 0.05); the lactic acid content (Figure 3C) of the NA group was significantly reduced 1 h and 3 h after feeding (*p* < 0.05); the activity of lactate dehydrogenase (Figure 3D) in the NA group was significantly reduced at 3 h and 5 h after feeding (*p* < 0.01); the NADH content (Figure 3F) in the NA group was significantly reduced (*p* < 0.01) at 3 h after feeding. There was no significant difference in other indicators between the two groups (*p* > 0.05). Other indicators between the two groups [pyruvate kinase (Figure 3B), NAD^+^ content (Figure 3E), succinic acid content (Figure 3G), succinate dehydrogenase activity (Figure 3H), malic acid content (Figure 3I) and malic acid dehydrogenase activity (Figure 3J)] was not significantly different at each time point (0 h before feeding, 1, 3, and 5 h after feeding) (*p* > 0.05).

### 3.4 Bacterial composition in the rumen

**Figure 4:**
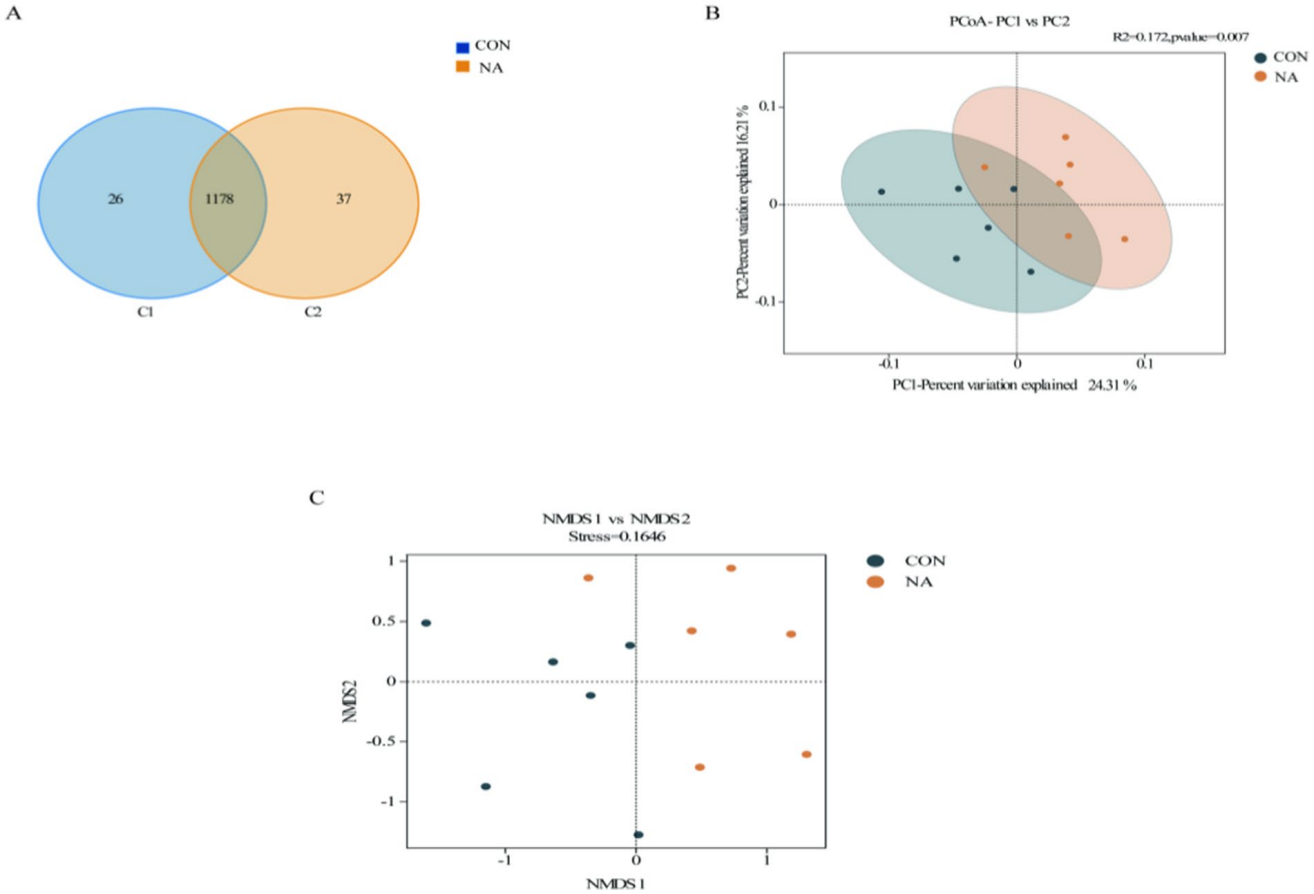
Rumen microbial analysis. CON, control group (basal diet); NA, niacin group (basal diet + 130 mg/day niacin). **(A)** Venn diagram of rumen microorganisms in the CON and NA (OTU). **(B)** Principal coordinate analysis (PCoA) of rumen microorganisms in the CON and NA. **(C)** Non-metric multidimensional scaling (NMDS) score plot of rumen microorganisms CON and NA.

**Figure 5:**
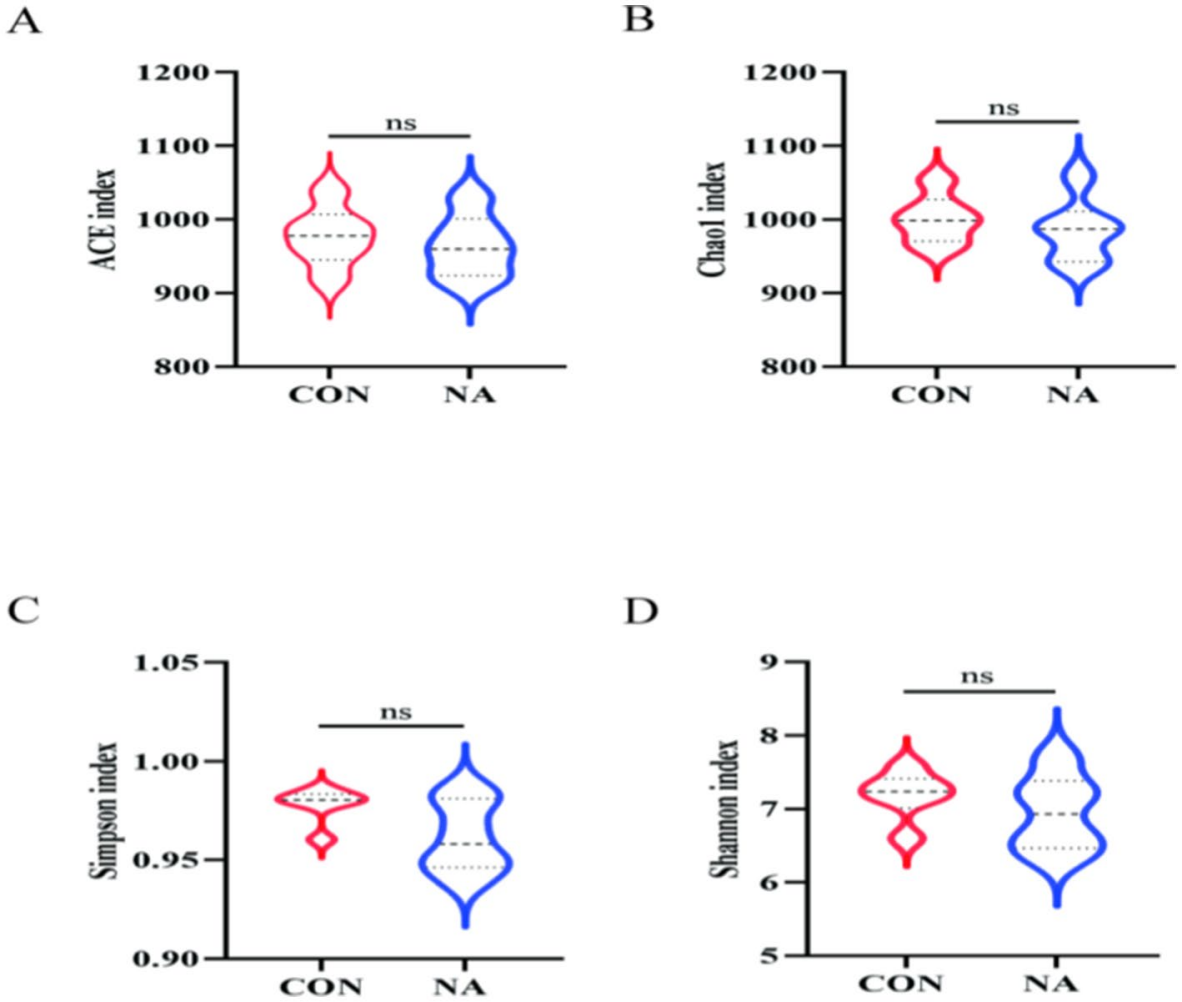
Rumen microbial analysis. CON, control group (basal diet); NA, niacin group (basal diet + 130 mg/day niacin), **(A–D)** α-diversity analysis of rumen microbial communities in sheep in the CON and NA.

In total, 959,870 raw reads were obtained from 12 samples. There were 930,233 effective reads, and the average length of the sequences was 419 bp. There were 26 and 37 OTUs unique to the CON and NA groups, respectively, and 1,137 OTUs common to both groups (Figure 4A). PCoA showed good separation of samples in each group (Figure 4B), with *R*^2^ and *p*-values of 0.712 and 0.007, respectively, and NMDS analysis further demonstrated significant differences between groups (Figure 4C). The Chao1, Ace, Shannon, and Simpson indices of the two groups were not significantly different (*p* > 0.05) (Figure 5).

**Figure 6:**
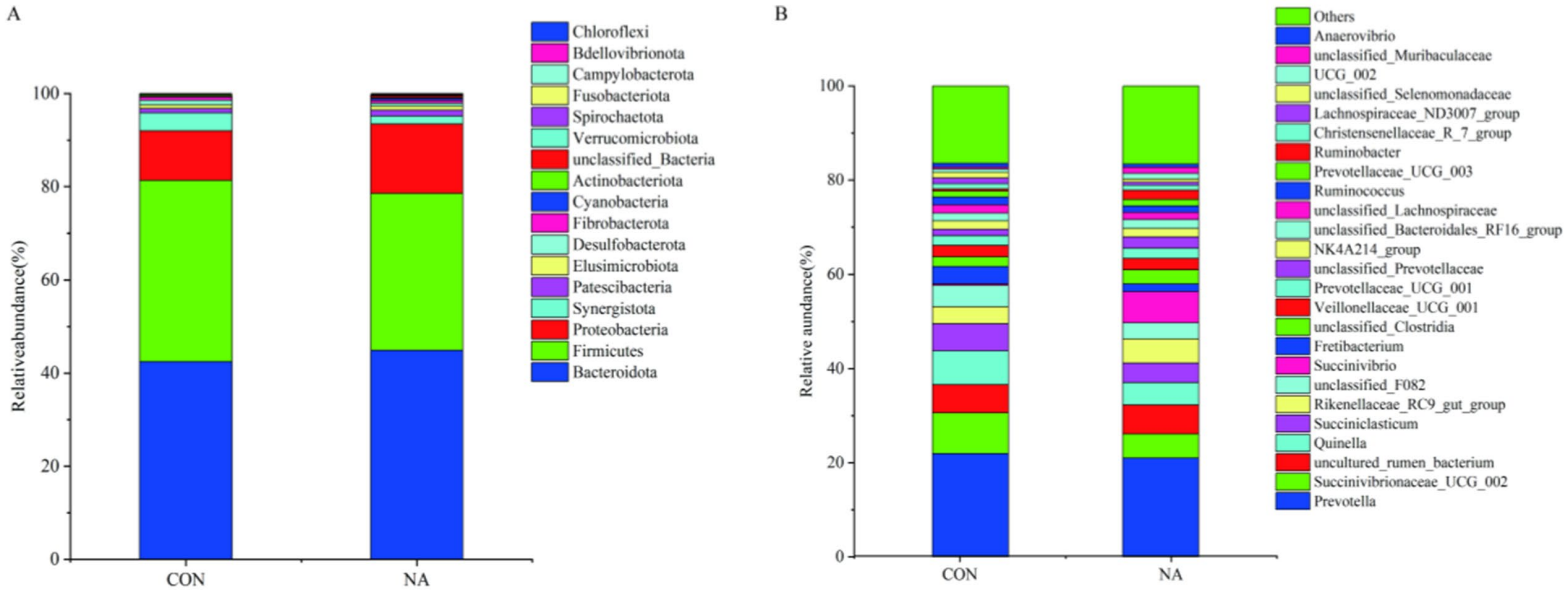
Rumen microbial analysis. CON, control group (basal diet); NA, niacin group (basal diet + 130 mg/day niacin). **(A)** Relative abundance of the phyla levels in the CON and NA. **(B)** Relative abundance of the genera levels in the CON and NA (as a percentage of the total sequence).

The taxonomic distribution of rumen microbiota at the phylum and genus levels is shown in Figure 6. Within the bacterial population, 18 phyla and 26 genera were identified in ruminal samples. Bacteroidetes was the predominant phylum, with a relative abundance of 43.70%, followed by Firmicutes (36.25%) and Proteobacteria (12.77%) (Figure 6A). At the genus level (Figure 6B), *Prevotella* (21.47%) was the predominant genera, followed by *Succinivibrionaceae*-UCG-002 (6.88%), and *Quinella* (5.95%).

### 3.5 Rumen metabolomic composition

**Figure 7:**
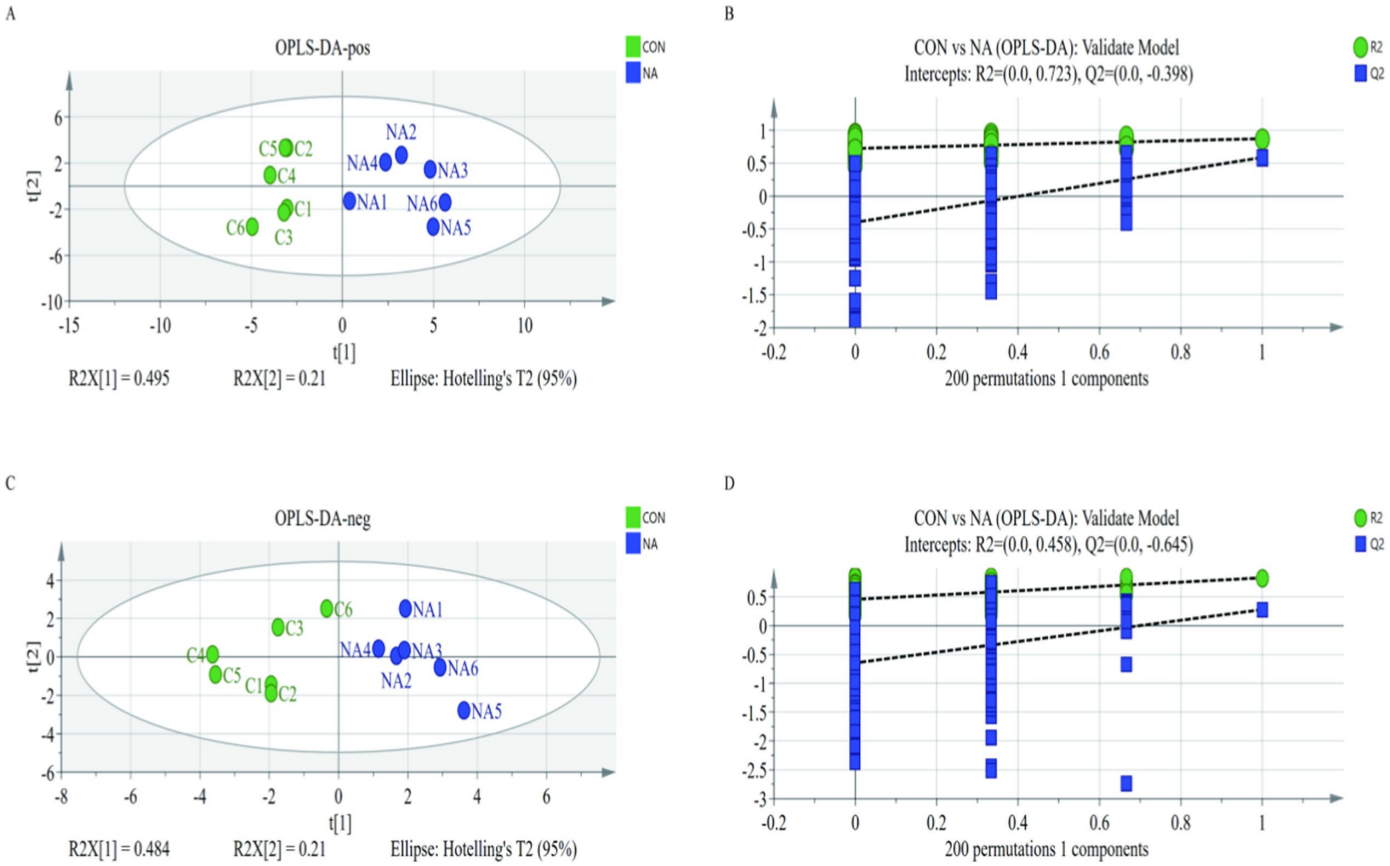
The orthogonal projection to latent structure-discriminant analysis model of the CON group **(A)** and the NA group **(C)**. Green dots and blue dots represent the permutation tests **(B,D)**. CON = C1, C2, C3, C4, C5, C6; NA = NA1, NA2, NA3, NA4, NA5, NA6. CON, control group (basal diet); NA, niacin group (basal diet + 130 mg/day niacin).

A PCA plot was used to visualize trends and outliers. The two groups were not separated when viewed using the first two principal components. To further examine metabolic changes, orthogonal projection to latent structure-discriminant analysis (OPLS-DA) was performed. The results showed that the *R*^2^*Y* and *Q*^2^*Y* of the ruminal fluids (positive and negative) were 0.705, 0.857, 0.695, and 0.28, respectively. The two groups were separated based on OPLS-DA score plots (Figures 7A,C). Additionally, the models showed good predictability, with no overfitting (Figures 7B,D).

### 3.6 Differential metabolite analysis

According to the statistical analysis and VIP values obtained by OPLS-DA, 831 metabolites were identified, and 36 specific differential metabolites were screened according to the principle: *p* < 0.05, (VIP) >1, (FC) value >1, or (FC) <1. Twenty-one of these metabolites were found to be at higher levels in the NA group than those in the CON group (*p* < 0.05), while 15 differential metabolites in the CON group were at higher levels than those in the NA group (*p* < 0.05). The compounds, *p*-value, VIP values, and FC values of each metabolite are shown in Table 3. The main metabolites differentially expressed between the CON and NA groups were amino acids, organic acids, sugars, and lipids. The levels of amino acids (such as L-histidine, norleucine, methylphenylalanine, aspartic acid, and phenylalanine), organic acids (such as glutaric acid, succinic acid, propionic acid, and L-malic acid), glucose and galactose (such as α-D-glucosamine-1-phosphate and 2-aminogalactopyranose), and lipids (o-cymene) in the NA group were higher than those in the CON group (*p* < 0.05). The levels of other amino acids (tetrahydropteroyl tri-L-glutamate), organic acids (maleic acid), glucose, galactose (chitobiose), and lipids (coumaric acid) in the NA group were lower than those in the CON group (*p* < 0.05).

**Table 3:** List of significantly different metabolites.

Compounds	*p*	VIP	FC
Amino acid			
2-(3-Carboxy-3-aminopropyl)-L-histidine	0.05	2.16	1.44
Norleucine	0.01	2.24	1.98
L-4-Hydroxy-3-methoxy-a-methylphenylalanine	0.02	1.95	2.28
Asp-Phe	0.02	2.04	1.80
Tetrahydropteroyltri-L-glutamic acid	0.01	2.31	0.62
Organic acid			
(N(omega)-L-arginino)succinic acid	0.03	1.96	1.08
Pyruvic acid	0.03	1.37	2.04
Glutaric acid	0.04	1.44	1.10
Acetic acid	0.03	0.37	1.07
Maleic acid	0.01	1.26	0.91
L-Malic acid	0.04	1.64	1.91
Glucose and galactose			
Octyl 2-acetamido-2-deoxy-alpha-D-glucopyranoside	0.04	1.73	1.38
2-Aminogalactopyranose	0.01	1.98	1.52
Alpha-D-glucosamine 1-phosphate	0.01	2.20	1.31
Chitobiose	0.04	1.89	0.58
2-Amino-2-deoxyglucitol-6-phosphate	0.03	1.89	0.53
Lipids			
o-Cymene	0.02	2.13	1.13
(R)-(-)-2-Phenylglycinol	0.02	1.97	1.55
Coutaric acid	0.04	1.68	0.45
Other			
Amobarbital	0.04	1.49	1.22
Neodunol	0.04	2.04	1.20
OPC4-CoA	0.02	1.99	8.28
2-Methylpyridine	0.02	2.07	1.17
1,5-Dideoxy-1,5-imino-D-galactitol	0.03	1.79	0.55
N-Palmitoyl-phosphoethanolamine	0.03	1.70	0.54
9S-Hydroxy-10E,12E-octadecadienoic acid	0.01	2.02	0.45
LIPC 18:0;3	0.04	1.74	0.45
{[(2S,3S,4R)-2-amino-3,4-dihydroxyicosyl]oxy}phosphonic acid	0.04	1.90	0.45
Capsi-amide	0.02	0.97	0.33
RO 40-5966 (Methylmibefradil Metabolite)RO 40-5966	0.03	1.94	0.45
KIRENOL	0.03	1.47	0.63
Sphinganine 1-phosphate	0.03	1.91	0.50
Ponasterone A	0.04	1.78	0.58
PC(P-15:0/0:0)	0.01	2.22	0.62
Cis,cis-2,4-dihydroxy-5-methyl-6-oxo-2,4-hexadienoate	0.01	1.89	0.55
S-Adenosylmethioninamine	0.03	1.69	0.28
P1-Uridyl-P2-phenyl diphosphate	0.03	2.17	0.33
2-Methoxyphenol	0.04	1.73	0.76
2-Pentylthiophene	0.04	1.86	0.61
(±)12,13-DiHOME	0.01	2.21	0.53
LysoPC(18:2(9Z,12Z))	0.02	1.89	0.43

VIP, importance projection of the OPLS-DA model; *p*, *p*-value of the *t*-test; FC, variance multiplier.

### 3.7 Metabolic pathways of differential metabolites

**Figure 8:**
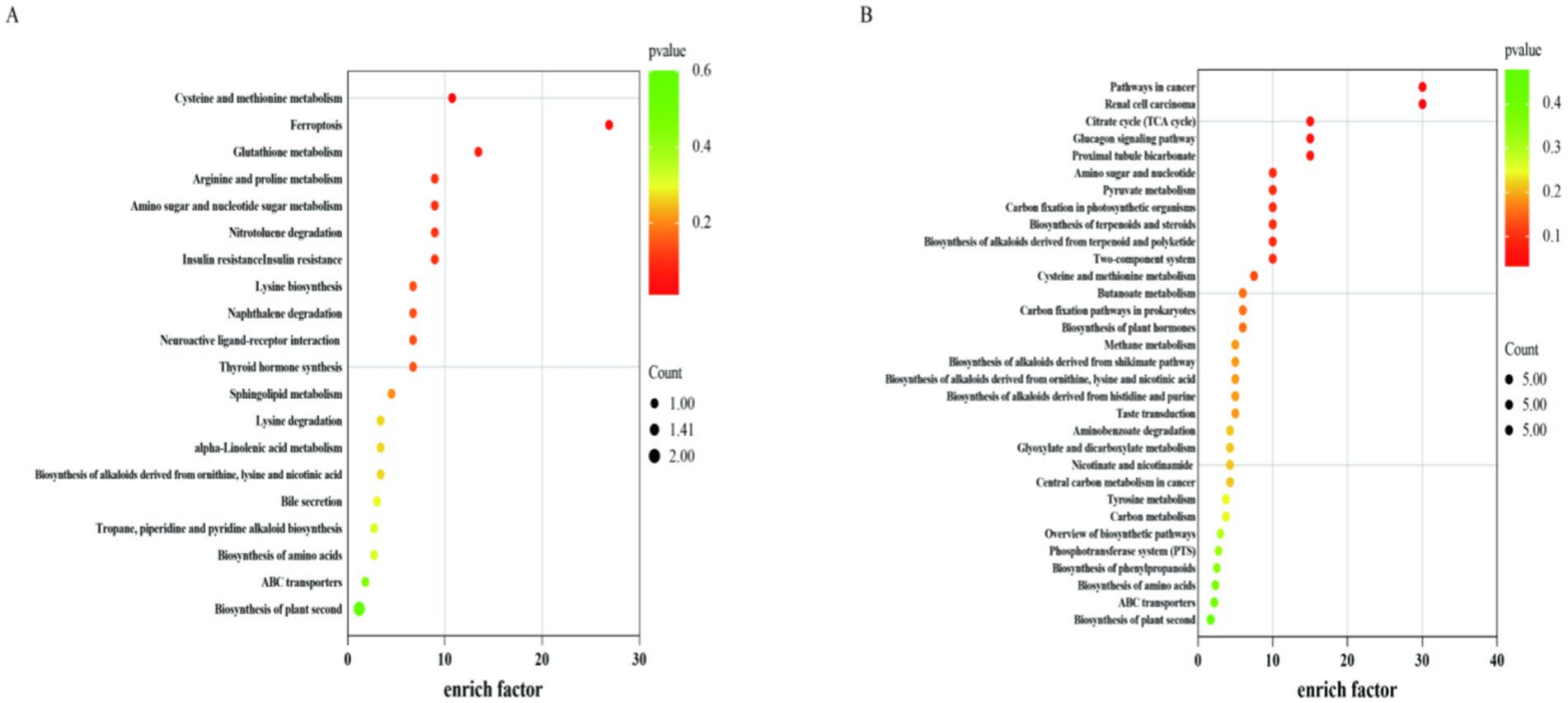
Metabolic pathway of the differential metabolite KEGG. CON, control group (basal diet); NA, niacin group (basal diet+130 mg/d niacin). The X-axis represents enrich fact and the Y-axis represents the pathway enrichment. The larger size of the circle indicates greater pathway enrichment and the darker color indicates higher pathway impact values. **(A)** Indicates KEGG pathway in positive ion mode in CON and NA groups; **(B)** Indicates KEGG pathway in negative ion mode in CON and NA groups.

According to the KEGG metabolic pathway display (Figure 8), there were 20 metabolic pathways for the positive ionization mode and 32 metabolic pathways for the negative ionization mode. This included pathways for the citric acid cycle, pyruvate metabolism, cysteine and methionine metabolism, glutathione metabolism, and arginine and proline metabolism.

### 3.8 Correlation analysis among rumen fermentation parameters, rumen microorganisms, and metabolome

**Figure 9:**
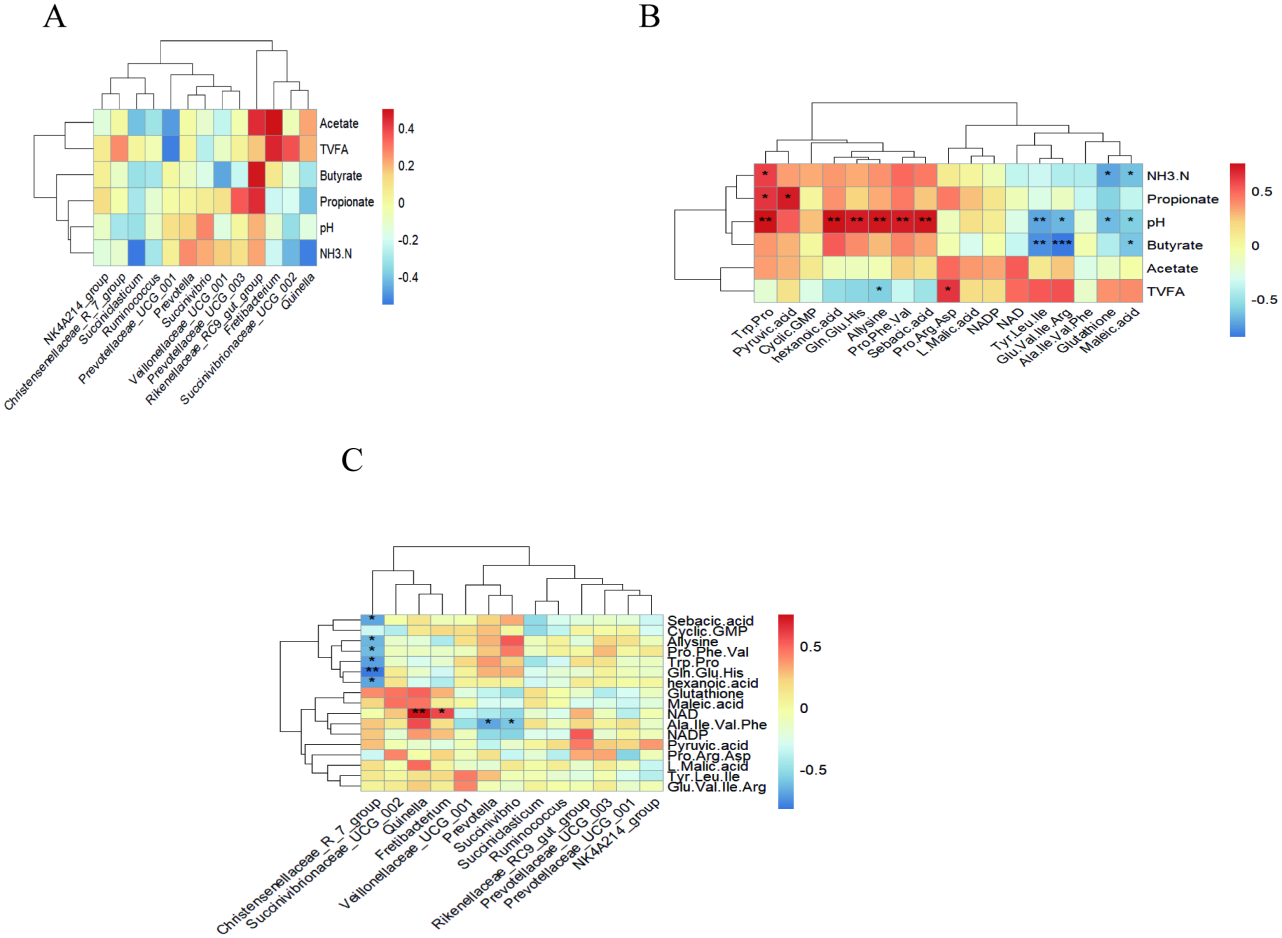
Heatmap showing the Spearman correlations between rumen fermentation parameters, microbial flora, and metabolites in the CON and NA. **(A)** Heat map of correlations between rumen fermentation parameters and microbial flora. **(B)** Heat map of correlations between rumen fermentation parameters and rumen metabolites. **(C)** Heat map of correlations between microbial flora and metabolites. A single asterisk (*) indicates a significant difference (*p* < 0.05), a double asterisk (**) or triple asterisk (***) indicate highly significant differences (*p* < 0.01).

Spearman’s correlation coefficient was used to calculate the correlation coefficient between each index (Figure 9). Correlation analysis between rumen fermentation parameters and rumen metabolites showed that NH_3_-N was positively correlated with Trp-Pro (*p* = 0.046, *r* = 0.594) and negatively correlated with glutathione (*p* = 0.415, *r* = −0.720), maleic acid (*p* = 0.409, *r* = −0.629). Propionate was positively correlated with Trp-Pro (*p* = 0.032, *r* = 0.350), pyruvic acid (*p* = 0.013, *r* = 0.706). pH was positively correlated with Trp-Pro (*p* = 0.005, *r* = 0.751), hexanoic acid (*p* = 0.005, *r* = 0.754), Gln-Glu-His (*p* = 0.010, *r* = 0.709), allysine (*p* = 0.005, *r* = 0.730), Pro-Phe-Val (*p* = 0.009, *r* = 0.716), and sebacic acid (*p* = 0.007, *r* = 0.726) and negatively correlated with Tyr-Leu-Ile (*p* = 0.010, *r* = −0.709), Glu-Val-Ile-Arg (*p* = 0.018, *r* = −0.667), glutathione (*p* = 0.023, *r* = −0.646), maleic acid (*p* = 0.042, *r* = −0.593). Butyrate was negatively correlated with Tyr-Leu-Ile (*p* = 0.004, *r* = −0.783), Glu-Val-Ile Arg (*p* < 0.001, *r* = −0.853), maleic acid (*p* = 0.035, *r* = −0.622). The total volatile fatty acids was positively correlated with Pro-Arg-Asp (*p* = 0.028, *r* = 0.629) and negatively correlated with allysine (*p* = 0.046, *r* = −0.594) (Figure 9B).

Correlation analysis between rumen microorganisms and rumen metabolites showed that *Christensenellaceae_R_7_group* was negatively correlated with sebacic acid (*p* = 0.017, *r* = −0.685), allysine (*p* = 0.026, *r* = −0.650), Pro-Phe-Val (*p* = 0.035, *r* = −0.622), Trp-Pro (*p* = 0.015, *r* = −0.699), Gln-Glu-His (*p* = 0.017, *r* = −0.825) and hexanoic acid (*p* = 0.016, *r* = −0.692). NAD was positively correlated with *Quinella* (*p* = 0.007, *r* = 0.755) and Fretibacterium (*p* = 0.046, *r* = 0.594). Ala-Ile-Val-Phe was negatively correlated with *Prevotella* (*p* = 0.017, *r* = −0.685), and *Succinivibrio* (*p* = 0.044, *r* = −0.589) (Figure 9C).

## 4 Discussion

When ruminants are fed highly concentrated rations for long periods, the rumen environment is often significantly altered, and ruminants may develop conditions, such as rumen acidosis. Niacin supplementation in buffalo diets can increase average daily weight gain (22). One study showed that dietary supplementation with 480–640 mg/kg niacin improved the growth performance of Jinjiang cattle (23). Supplementing the diet with niacin can increase NAD^+^ and NADH levels in the rumen, which significantly improves the growth and development of rumen microorganisms, promotes the growth and fattening of animals, and improves productivity (24). In the present study, the DMI was significantly increased in the NA group compared to CON group, the concentration of synthetic niacin in the rumen of the CON group was probably low and did not meet the nutritional needs of the animals. During the whole experimental period, the final BW, DMI, and ADG of the CON group did not increase significantly as that of the NA group. The level of NAD^+^ in the rumen of the NA group increased, promoting the growth of rumen microorganisms and improving the utilization of nutrients and growth performance of sheep (25).

In this study, the pH of the CON group and the NA group were between 5.83–6.22 and 5.87–6.18, respectively, indicating that niacin can alleviate rumen acidosis by inhibiting the proliferation of acid-producing bacteria (*Streptococcus bovis*), producing more NAD^+^, inhibiting the activity of lactate dehydrogenase, and reducing the production of lactic acid (9, 26). These results are similar to those of the studies cited above. Niacin shortens the duration of low pH under high-concentrate diet conditions.

The concentration of NH_3_-N in the rumen reflects the degradation of proteins in the feed and is the main nitrogen source for rumen microorganisms to synthesize microbial proteins (27). In the present study, the concentration of ammonia nitrogen increased in the NA group. After entering the rumen, niacin provided a sufficient nitrogen source and promoted the degradation of protein in feed by rumen microorganisms, consistent with the results reported by Zhang (28).

After entering the rumen, carbohydrates are degraded by rumen microorganisms into volatile fatty acids (mainly acetic, propionic, and butyric acids), which are the main energy sources for ruminants (29). Its concentration is related to the ratio of dietary concentrate to coarse grains. Propionic acid and butyric acid are the main sources in the ruminal epithelium. This study showed that the concentrations of propionic acid and butyric acid significantly increased in the NA group. It can be seen that propionic acid-type fermentation provides higher energy to the body. Niacin increases the abundance of propionic and butyric acid-producing bacteria (*Succinivibri*o and *Crescentomonas ruminantium*) in the rumen and promotes the production of fermentation substrates, thereby increasing the concentrations of propionic and butyric acids.

Ruminal lactate metabolism-related enzymes play a key role in the conversion of intermediate metabolites of ruminal lactate into final products and can accelerate the conversion of intermediate metabolites. The succinate pathway is one of the main processes involved in carbohydrate metabolism in ruminants. Pyruvate is catalyzed by enzymes, such as pyruvate carboxylase, malate dehydrogenase, and succinate dehydrogenase, to produce oxaloacetate, malate, succinate, and propionate (30). This study showed that the NA group had increased pyruvate, succinate, and malate contents, as well as increased activities of pyruvate kinase, NAD^+^, succinate dehydrogenase, and malate dehydrogenase, and decreased lactic acid, lactate dehydrogenase, and NADH contents. Under the catalysis of malate dehydrogenase and succinate dehydrogenase, malate and succinate stimulate ruminant *Crescentomonas* to utilize lactate and produce propionic acid (31).

Changes in the rumen microbial community affect rumen fermentation and the metabolic levels of animals. According to the PCoA and NMDS analyses, the CON and NA group samples were separated, indicating that niacin improved rumen microbial community structure to a certain extent. Shannon and Simpson indices reflect species diversity, whereas ACE and Chao1 indices mainly measure species richness (32). Although there was no significant difference in each index between the two groups, the NA group showed higher species diversity and richness than the NA group, and the high richness and diversity of the bacterial community were considered beneficial and could increase rumen stability (33). The α-diversity results further demonstrated that niacin changed the rumen microbial diversity.

In this study, the relative abundance of *Prevotella* showed a strong and significant negative correlation with pH. The relative abundance of *Prevotella* was lower in the NA group, while the pH was significantly higher in the NA group. It is possible that niacin shortened the duration of low pH in high-concentrate feeds and affected the relative abundance of *Prevotella*. This is consistent with the findings of Luo et al. (34) and Zhang et al. (35). The relative abundance of *Prevotella* was positively correlated with the total VFA concentration, consistent with the results of Khafipour et al. (36).

In this trial, Firmicutes, Bacteroidetes, and Proteobacteria were the dominant phyla in rumen microorganisms, consistent with other research results (37, 38). The relative abundances of Proteobacteria and Bacteroidetes in the NA group were higher than those in the CON group, whereas the relative abundance of Firmicutes in the NA group was lower than that in the CON group. This may be because the addition of niacin inhibited the growth of gram-positive bacteria (Firmicutes), causing the relative abundance of Firmicutes to decrease, while promoting the growth of gram-negative bacteria (Proteobacteria and Bacteroidetes). The phylum-level distribution showed that the richness and uniformity of the microbial flora in the CON group were lower than those in the NA group.

Ruminants rely on starch-, cellulose-, and hemicellulose-degrading bacteria to digest and utilize these carbohydrates in the feed. *Prevotella*, belonging to the phylum Bacteroidetes, plays an important role in the degradation of starch, cellulose, and protein; directly participates in the digestion of carbohydrates and changes the rumen fermentation patterns (39, 40); and mainly produces acetic acid, succinic acid, and propionic acid, of which propionic acid is primarily synthesized through the acrylic acid pathway (41). *Quinella* spp. are mainly involved in carbohydrate metabolism (42). *Succinivibrio* are common ruminal bacteria. They participate in the metabolism of animals to produce acetic, propionic, and succinic acids. Studies have shown that niacin can increase the relative abundance of succinic acid, indicating that it can promote the production of propionic acid in high-concentrate feeds (43). In this experiment, the relative abundances of *Prevotella*, *Succinivibrio*, and *Quinella* changed significantly. The relative abundances of *Prevotella* and *Quinella* in the NA group were lower than those in the CON group, whereas the relative abundances of *Succinivibrio* and Butadiene were higher than those in the CON group. This indicates that niacin addition can metabolize carbohydrates, reduce the production of acetic acid to a certain extent, increase the synthesis of propionic acid, and continue the energy metabolism process to provide energy for ruminants.

Metabolomics revealed differences in rumen metabolites between the CON and NA groups of sheep. The experiment not only studied the differential metabolites between the CON and NA groups but also analyzed the metabolic pathways in which these metabolites were involved. The *p*-value was calculated using KEGG enrichment analysis, and the key metabolic pathways were explored based on the VIP and *p*-value (44–46). The main metabolic pathways included sucrose and starch, tryptophan, vitamin B6, and propionic acid metabolism. Significant differences in the metabolic pathways involved L-malic acid and succinic acid, indicating that they play a vital role in the succinic acid metabolic pathway. L-malic acid and succinic acid are key intermediate metabolites that correspond to these results. The results above showed that the activities of succinate dehydrogenase and malate dehydrogenase in the rumen increased, and the succinate and malate contents increased as well. The results of the ruminal microbial research showed that the relative abundance of the genus *Succinivibrio* increased. *Succinivibrio* can use fumaric acid and malic acid to produce succinic acid, which is then converted to succinyl-CoA by succinate dehydrogenase to produce propionic acid. The levels of L-malic acid, succinic acid, and propionic acid in the NA group were higher than those in the CON group, especially L-malic acid (*p* = 0.04, FC = 1.91, VIP = 1.64), succinic acid (*p* = 0.03, FC = 1.08, VIP = 1.96), and propionic acid (*p* = 0.03, FC = 2.04, VIP = 1.37). These results indicated that niacin improves energy metabolism by regulating the intermediates in the succinic acid metabolic pathway.

Correlation analysis among rumen fermentation parameters, rumen microorganisms, and metabolites showed that propionic acid was correlated with *Prevotellaceae_UCG_003*, further demonstrating the importance of niacin in high-concentrate feeding.

## 5 Conclusion

In this experiment, adding 130 mg/day of niacin to a high-concentrate diet had a good effect on alleviating the symptoms of rumen acidosis. Niacin can affect metabolites by changing rumen fermentation conditions and rumen microorganisms, thereby improving animal production performance, including increasing dry matter intake, increasing rumen pH, and improving rumen fermentation function. During the fattening period, a high proportion of concentrate feed may lead to rumen acidosis. Therefore, adding niacin to the feed can effectively help cattle and sheep avoid rumen acidosis during the fattening period.

## Data Availability

The datasets presented in this study can be found in online repositories. The names of the repository/repositories and accession number(s) can be found below: NCBI, PRJNA1168411.

## Footnotes

^1^ https://mothur.org/wiki/454_sop/
